# Degradation of Albumin on Plasma-Treated Polystyrene by Soft X-ray Exposure

**DOI:** 10.3390/polym8070244

**Published:** 2016-06-24

**Authors:** Nina Recek, Gregor Primc, Alenka Vesel, Miran Mozetic, José Avila, Ivy Razado-Colambo, Maria C. Asensio

**Affiliations:** 1 Jozef Stefan Institute, Jamova cesta 39, Ljubljana 1000, Slovenia; Nina.Recek@ijs.si (N.R.); Gregor.Primc@ijs.si (G.P.); alenka.vesel@ijs.si (A.V.); miran.mozetic@guest.arnes.si (M.M.); 2 Synchrotron-SOLEIL & Université Paris-Saclay, Saint-Aubin, BP48, Gif sur Yvette Cedex F91192, France; jose.avila@synchrotron-soleil.fr (J.A.); ivy.razado-colambo@synchrotron-soleil.fr (I.R.-C.)

**Keywords:** soft X-rays, radiation damage, albumin, HSA, NEXAFS, XANES

## Abstract

Thin films of human serum albumin (HSA) were immobilized on polystyrene (PS) substrates previously functionalized either with polar or nonpolar functional groups. The functionalization was performed by treatment with cold gaseous plasma created in pure oxygen and tetrafluoromethane (CF_4_) plasmas, respectively. Samples were examined with soft X-rays in the photon energy range of 520 to 710 eV in the ANTARES beam line at SOLEIL Synchrotron. NEXAFS spectra of O K-edge and F K-edge were collected at different spots of the sample, and measurements at each spot were repeated many times. A strong degradation of the HSA protein was observed. The weakly irradiated samples exhibited strong absorption at 531.5 eV associated with the O 1s→π*_amide_ transitions, and a broad non distinctive peak at 540 eV was attributed to the O 1s→σ*_C–O_ transitions. Both peaks decreased with increasing irradiation time until they were completely replaced by a broad non-distinctive peak at around 532 eV, indicating the destruction of the original protein conformation. The shortage of the amide groups indicated breakage of the peptide bonds.

## 1. Introduction

The behavior of medical implants upon contact with body liquids, cells and tissues is currently a hot topic in multidisciplinary science [1]. The interaction mechanisms are extremely complex such that they are far from being well understood despite previous in vivo tests performed decades ago. Presently, insights into the mechanisms are only possible using in vitro tests with selected materials. Typically, a substrate or scaffold is prepared from polymers and incubated with model biological materials. Both types of materials are carefully characterized using available techniques and the interaction mechanisms are studied during and after incubation. A good example of such studies is the interaction of cardiovascular implants with blood. The goal of such studies is to develop novel vascular grafts, stents, and heart valves with improved hemocompatible properties [2,3]. Current available products do not exhibit optimal biocompatibility as reflected in the uncontrolled adsorption of blood constituents, the activation of blood platelets, and inadequate endothelization. These effects may lead to formation of thrombus and/or to bypass graft failure.

One possible method to improve polymer graft hemocompatibility is modification of surface properties by treatment with gaseous plasma [4,5,6,7]. Polymers are exposed to reactive gaseous particles found in glowing plasma or its afterglow for the following reasons: (i) cleaning; (ii) functionalization; (iii) nanostructuring; and (iv) sterilization. The cleaning efficiency of plasma radicals has been known for decades. Oxygen and/or nitrogen radicals are used to remove traces of organic impurities from polymer surfaces [8]. Plasma radicals also interact chemically with the surface of polymers causing formation of new functional groups. In the case of oxygen plasma treatment, carboxyl, hydroxyl and carbonyl groups are formed on polymer surfaces. Nitrogen plasma is applied in order to create amine and amide groups [9] while fluorine-containing groups are found on polymer surfaces after treatment with C*_x_*F*_y_* gases, typically CF_4_ [10,11]. The thickness of the modified films on polymer surfaces depends on the plasma parameters and is typically a few nanometers. The bulk properties of plasma-treated polymers remain fairly intact as long as the treatment is performed at room temperature and the samples are not irradiated with energetic particles, such as VUV radiation and fast ions formed in plasma, which are generated with some types of gaseous discharge. These unwanted effects are efficiently avoided by using flowing afterglows, which are free from energetic ions and UV radiation, instead of the plasma itself, to modify the polymer surface properties [12]. The functionalization is often accomplished in a very short treatment time (less than a second); then, the next process is etching of polymers [13]. Etching is usually inhomogeneous due to small variations in the structure of polymer materials. Amorphous component is etched less intensively than the crystalline phase. In the case of co-polymers, there is always a difference in etching rates for the components. Thus, etching causes nanostructuring of the polymer morphology [9]. Finally, a consequence of plasma treatment is also sterilization, i.e., destruction of bacteria, viruses, fungi and alike that might be present on the surface of the as-received polymer product. Sterilization occurs due to various effects such as exposure to unpleasant vacuum conditions, chemical etching, UV destruction, localized thermal effects, and electrostatic effects [14,15]. In any case, plasma treatment allows a good preparation of the substrates prior to biological experiments. In many cases, the biological response of plasma-modified materials for vascular grafts is improved dramatically since such treatments prevent activation of blood platelets [16]. Biological tests are definitely suitable for studying the hemocompatibility of cardiovascular implants, but do not reveal the mechanisms that cause such observations. Therefore, more fundamental studies are needed.

State-of-the-art and advanced analytical techniques for material characterization are available at modern synchrotrons such as X-ray absorption spectroscopy, photoelectron spectroscopy and X-ray diffraction. Synchrotron radiation-based techniques proved superior as compared to techniques based on standard X-ray sources for studying inorganic samples. Although these techniques are widely used to study complex organic materials [17,18,19,20,21,22], proper precautions in performing such experiments should be considered since organic materials are delicate and their properties could change upon irradiation. Such damage has been reported for a variety of materials including poly methyl methacrylate [23,24], halogen-containing polymers [25,26], cellulose [27].

A major effect governing the behavior of vascular grafts made from polymer materials is the adsorption of blood proteins, especially human serum albumin (HSA); which is a major blood component. This phenomenon was studied using NEXAFS (Near-edge X-ray absorption fine structure) spectroscopy, by various authors [18,19]. Thus far, soft X-ray irradiation damage in albumins has not been reported. In order to address the possible changes in albumin properties during irradiation with synchrotron light, we performed systematic experiments using various techniques. The results clearly indicate huge changes in the albumin structure during NEXAFS measurements.

## 2. Materials and Methods

### 2.1. Polystyrene Substrates

Polystyrene (PS) foils with a thickness of 0.25 mm were purchased from Goodfellow Ltd. (Huntingdon, UK). PS is a synthetic thermoplastic polymer with negligible biodegradability. It is widely used in biology, for example, as a substrate for cell proliferation. The polymer was cut into several disks with a diameter of 1 cm and cleaned in an ultrasonic bath using 100% EtOH.

### 2.2. Human Serum Albumin

The polymer substrates were incubated in an aqueous medium containing 1% protein human serum albumin (HSA) purchased from Sigma-Aldrich Co*.,* (Taufkirchen, Germany). HSA is the most abundant protein in human blood plasma. Samples of PS polymer were incubated in a 1% solution of HSA for 1000 s in order to allow stable conformation. After incubation, the samples were rinsed with water and left to dry.

### 2.3. Gaseous Plasma Treatment

Samples of PS foils were treated with highly reactive gaseous plasma created in oxygen or CF_4_ gas in order to modify their surface properties. Plasma was created in a 50-cm-long glass discharge tube with a 4 cm diameter. The discharge tube was pumped with a two stage rotary pump and the gas was leaked into the tube on the other side. Continuous pumping and gas leakage established the pressure of 50 Pa in the discharge tube. Plasma was created along the tube using electrodeless radiofrequency discharge. A copper coil mounted onto the tube was connected to a RF generator via a matching network. The generator operated at the standard industrial frequency of 13.56 MHz and the output power was set to 200 W. Under such discharge conditions, gaseous molecules in the plasma dissociate well upon inelastic collisions with plasma electrons, and atoms do not associate with parent molecules due to the following reasons: (i) gas pressure is low enough to prevent three body collisions needed for gas phase recombination; and (ii) quartz glass is rather inert to plasma particles at room temperature, thus the probability for heterogeneous surface recombination of atoms to parent molecules is low. Neutral atoms formed with electron-induced dissociation of parent molecules therefore abound in plasma and interact chemically with the polymer surface forming specific functional groups. The plasma treatment time was 60 s.

### 2.4. Atomic Force Microscopy (AFM)

An AFM (Solver PRO, NT MDT, Moscow, Russia) was used to characterize the surface topography of the samples. All measurements were done under semi contact mode using golden silicon probes NSG10 tips (NT MDT, Limerick, Ireland) with a resonance frequency of 140–390 kHz and a force constant of 3.1–37.6 N/m.

### 2.5. X-ray Photoelectron Spectroscopy (XPS)

Samples were analyzed by a high-resolution XPS instrument «TFA XPS Physical Electronics» (Physical Electronics Inc., Chanhassen, MN, USA). The base pressure in the XPS analysis chamber was about 6 × 10^−8^ Pa. The samples were excited with monochromatic Al K_α1,2_ radiation at 1486.6 eV over an area of 400 µm^2^. Photoelectrons were detected with a hemispherical analyzer positioned at an angle of 45° with respect to the normal of the sample surface. XPS survey spectra were measured at a pass energy of 187 eV using an energy step of 0.4 eV, while high-resolution C1s spectra were measured at a pass energy of 23.5 eV using an energy step of 0.1 eV. An additional electron gun was used for surface neutralization during XPS measurements. The measured spectra were evaluated using MultiPak v8.1c software (Ulvac-Phi Inc., Kanagawa, Japan, 2006), which was supplied with the spectrometer.

### 2.6. Near-Edge X-ray Absorption Fine Structure (NEXAFS)

X-ray absorption spectra were recorded at the ANTARES beam line specially designed in performing complementary photoemission and soft X-ray absorption spectroscopy, at the SOLEIL Synchrotron, under ring operating conditions of 2.5 GeV electron energy, an injection current of 500 mA and “Top-up” mode. The radiation was monochromatized using a plane-grating monochromator (PGM), which is characterized by a slitless entrance and the use of two Varied Linear Spacing (VLS) gratings with a Variable Groove Depth (VGD) along the grating lines. All measurements were made at 20 (±0.2) °C and 1.2 × 10^−8^ Pa. Data were collected over the range of 520–570 eV and 670–710 eV, for the O K-edge and F K-edge, respectively, permitting a correct normalization of the XANES spectra with 0.5 eV steps over the regions covering the edges. After data collection, the spectra were normalized to the incident intensity *I*_0_, which was obtained by collecting the total electron yield intensity from a gold-coated 90% transmitting grid placed in the incoming X-ray beam path. Moreover, the photon beam illuminated the sample surface at an incident angle of 45° with respect to the sample normal, in order to avoid any uncontrolled polarization NEXAFS dependence [28,29].

A precise determination of the electronic structure of the polystyrene with and without albumin and previously treated with cold gaseous plasma produced in pure oxygen and CF_4_ was carried out by high resolution XANES of the O K-edge and F K-edge using Total Fluorescence Yield (TFY) detection. Moreover, successful recording of experimental NEXAFS O K-edge spectra was achieved during the albumin degradation upon controlled illumination doses with soft X-ray.

## 3. Results and Discussion

### 3.1. AFM Imaging

**Figure 1:**
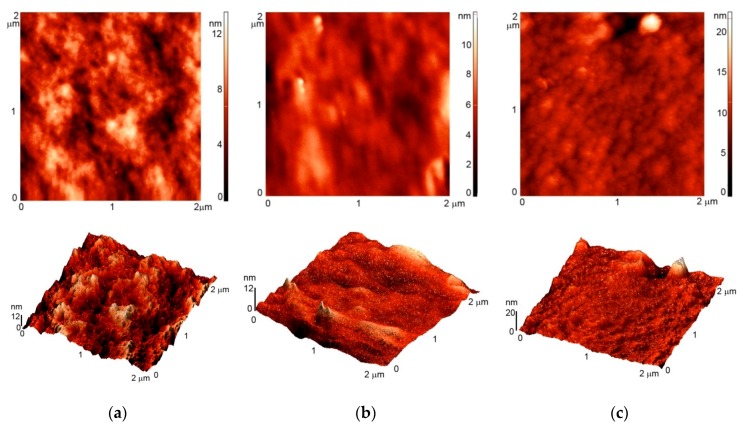
AFM images of: (**a**) untreated sample; (**b**) sample treated in oxygen plasma; (**c**) sample treated in CF_4_ plasma.

**Figure 2:**
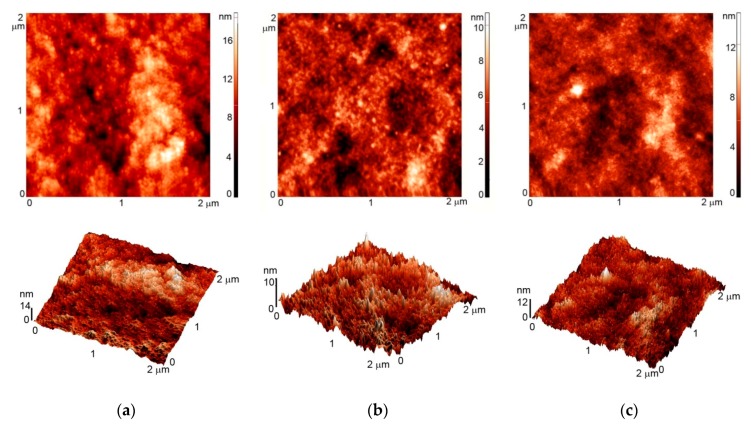
AFM images of: (**a**) untreated sample coated with albumin; (**b**) sample treated in oxygen plasma and coated with albumin; (**c**) sample treated in CF_4_ plasma and coated with albumin.

Typical AFM images of untreated polystyrene samples are presented in Figure 1. The original material is rather smooth since no specific morphological features are observed. The average roughness deduced from the AFM image is 1.5 nm. Plasma treatment causes slight increase of the roughness to 2.8 and 3.5 nm for oxygen and CF_4_ plasma treatment, respectively. Figure 2 represents the images for PS samples after incubation with albumin. The images do not reveal formation of agglomerates or any other features that might form on surfaces upon inappropriate incubation. Nevertheless, the surface roughness was increased after incubation with albumin, i.e., 3.6, 9.2 and 7.9 nm for untreated, oxygen-plasma treated and CF_4_ plasma-treated samples, respectively.

### 3.2. XPS Results

**Figure 3:**
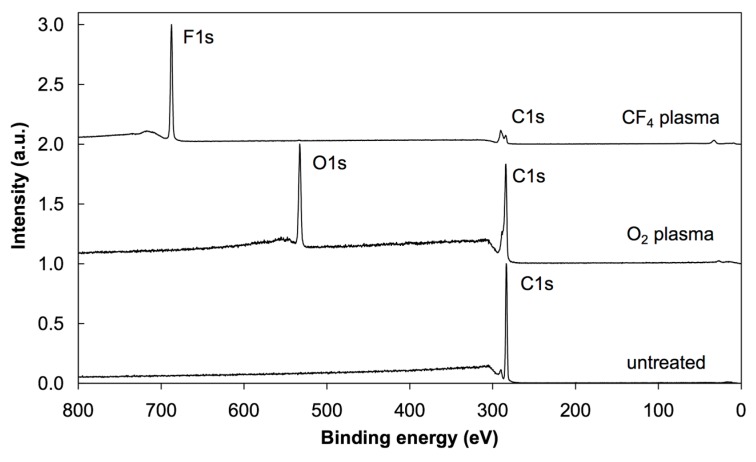
XPS overview spectra of untreated, oxygen and CF_4_ plasma-treated PS samples.

**Figure 4:**
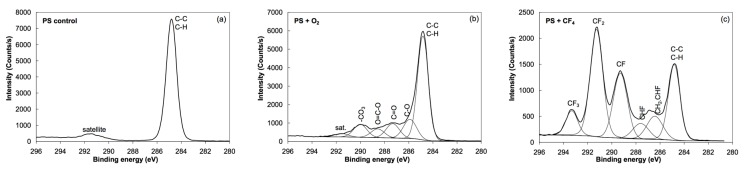
High-resolution C 1s peaks of: (**a**) untreated PS sample; (**b**) oxygen plasma-treated PS sample; (**c**) CF_4_ plasma-treated PS samples.

Photoelectron spectra were acquired at different spots on the samples to study the reproducibility of the results. Minimal variations were observed, indicating rather uniform films on the surface of all samples in agreement with the AFM results. XPS characterization was performed using a conventional instrument since any attempt to apply synchrotron radiation failed due to strong charging of the surface upon irradiation with soft X-rays. The energy resolution using conventional XPS was sufficient to obtain high-resolution C 1s spectra. Figure 3 shows the overview spectra of untreated, oxygen- and CF_4_-plasma treated PS samples. For the untreated sample (bottom curve), only the C 1s peak appeared, which implies that the PS material does not adsorb any contaminant. The absence of oxygen indicates that the PS does not interact with air under ambient conditions. Plasma treatment, on the other hand, allows functionalization with specific functional groups. The overview spectra (middle and upper curves) show strong functionalization since the concentration of oxygen is about 27 at %, while that of fluorine is 56 at %. The concentration of the elements as revealed in the overview spectra is summarized in Table 1. Figure 4 displays the high resolution C 1s spectra of the untreated and plasma-treated PS samples. Identification of the specific functional groups could be deduced from these spectra. Untreated samples reveal only C–C and C–H bonds, and a well-defined shakeup feature at 291.5 eV characteristic of the aromatic ring. Upon oxygen plasma treatment, different oxygen functional groups are revealed and the satellite peak is smaller, indicating breakage of the aromatic ring [13]. Such rich functionalization is explained by the interaction of reactive oxygen particles with the otherwise oxidation resistant polymer. The surface becomes extremely hydrophilic and thus the functional groups represent anchor sites for polar functionalities likely to be found in albumin. In the case of CF_4_ plasma treatment, different bonds between the C and F atoms are clearly observed in Figure 4. Any destruction of the aromatic rings could not be deduced from this figure due to the overlap of the satellite carbon peak with the –CF_3_ and –CF_2_ peaks. The richness of fluorine indicates almost perfect fluorination suitable for adsorption of albumin due to hydrophobic interactions, which should, according to the literature [30,31,32], allow strong adhesion and reconformation of proteins on such surfaces [33,34,35].

**Figure 5:**
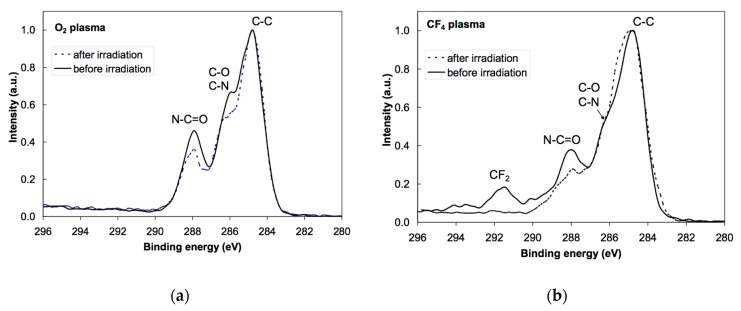
High resolution C 1s peaks prior and after NEXAFS analyses of: (**a**) oxygen plasma-treated PS sample incubated in albumin solution; (**b**) CF_4_ plasma-treated PS sample incubated in albumin solution.

**Figure 6:**
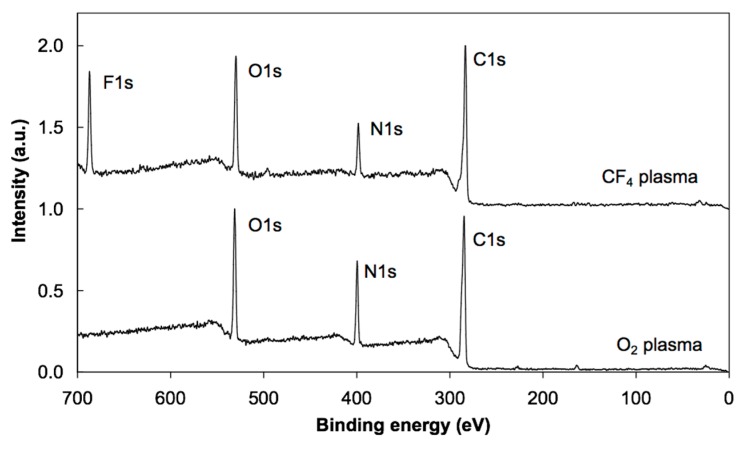
XPS overview spectra of PS samples treated in oxygen or CF_4_ plasma and incubated in albumin solution prior to NEXAFS analyses.

The high resolution C 1s peak for oxygen plasma-treated sample incubated in albumin solution is presented in Figure 5. No peak deconvolution was performed due to a large diversity of functionalities present in albumin. Still, a huge difference between just functionalized (Figure 4) and functionalized samples after incubation in protein solution (Figure 5) is revealed. The absence of the –CO_3_ peak indicates total coverage of the oxygen plasma-treated PS with albumin. This observation corroborates with the existing hypotheses on the interaction of blood proteins with highly hydrophilic polymer surfaces in which a thick multilayered adsorption occurs. This is explained by the specific conformation of albumin on such surfaces, which results in a thick coating [36,37,38,39,40]. The overview spectrum in Figure 6 supports this conclusion since the intensity of the N peak is similar to that of the pure albumin. Thus, the oxygen observed in the overview spectrum originates from the albumin itself and not from the oxygen-rich interface between the polymer and the albumin formed upon treatment with oxygen plasma.

The situation is different after incubating the sample treated with CF_4_ plasma. Aside from the persistence of F 1s peak, the shape of the C 1s peak is completely different from the oxygen plasma-treated sample (Figure 5). This is explained by the different conformation of the albumin on hydrophobic surfaces. Instead of a thick film, a monolayer of albumin is deposited upon incubation of the CF_4_ plasma treated sample. That is why fluorine rich peaks are observed both in the overview spectrum in Figure 6 and in the high resolution C 1s peak in Figure 5. In particular, the peak at 291.5 eV in Figure 5 fits with the CF_2_ peak in Figure 4.

**Figure 7:**
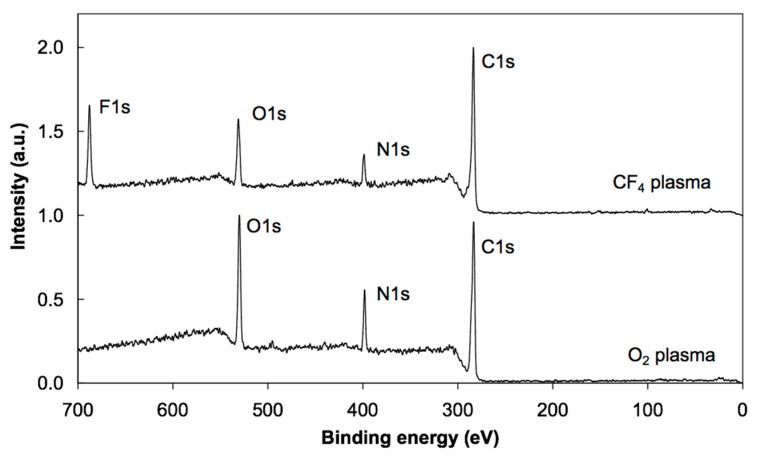
XPS overview spectra of PS samples treated in oxygen or CF_4_ plasma and incubated in albumin solution after NEXAFS analyses.

In Figure 5, C1s spectra of the oxygen and CF_4_ plasma-treated samples, before (solid line) and after (dashed line) irradiation with X-rays are shown. Obvious differences in the spectra could be observed before and after irradiation. These variations are reproducible when probing different spots on the sample and well above the detection limit of the XPS technique. The reasons will be discussed in the next subsection, also taking into account the survey spectra obtained after irradiation (Figure 7).

### 3.3. NEXAFS Analyzes

While XPS and UPS can be exploited to study the occupied density of states in a material, Near-edge X-ray Absorption Fine Structure (NEXAFS) is a spectroscopic tool which provides complementary information on the unoccupied states, often called X-ray Absorption Near-Edge Spectroscopy (XANES). This technique gives the best results using highly brilliant radiation available at third generation synchrotron facilities. Soft X-ray radiation is typically used to probe the absorption K-edge of low atomic number elements such as C, F, O and N. The technique has the advantage that if the photon energy is not sufficient to eject an electron from the sample into the vacuum but instead corresponds to the transition energy between a core level (e.g., the 1s level) and an unoccupied level of a molecule (e.g., the LUMO), the core electrons can be resonantly excited into an unoccupied bound molecular orbital. These transitions give rise to characteristic resonances close to the absorption edge (the so-called ‘‘fine structure’’). In a typical NEXAFS measurement, the photon energy is scanned across the absorption edge of the chosen element while the corresponding absorption is measured; therefore, the use of tunable synchrotron radiation is required. Identifying the transition energies of the spectra, detailed information can be gained on the unoccupied electronic states of the sample. Similar to XPS, NEXAFS is elemental-sensitive and the absorption fine structure is strongly dependent on the chemical and structural environment of the atoms present in the sample investigated.

**Figure 8:**
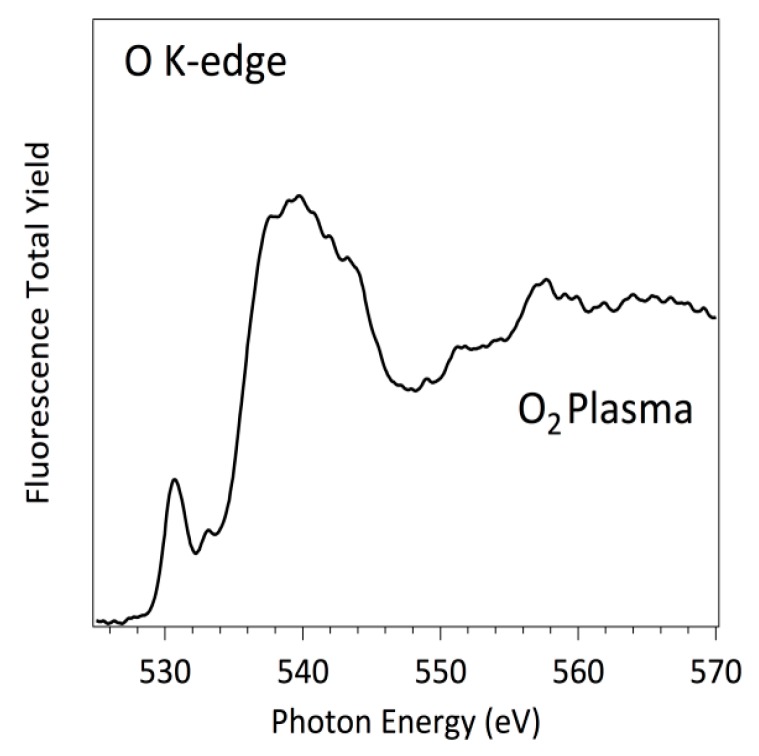
O K-edge NEXAFS spectrum of PS samples treated with oxygen plasma.

Firstly, the polystyrene surface treated with O_2_ plasma was investigated using O K-edge NEXAFS measurements (Figure 8). As it has been mentioned, NEXAFS relies on a polarized beam of high-intensity X-ray from a synchrotron source, which excites a core electron to create a core hole [41,42,43]. This hole is then filled by an outer-shell electron, and the excess energy causes the emission of a fluorescence photon or an Auger electron. For the K and L shells of low-Z elements, electron decay is more probable than fluorescence and therefore has a much favorable signal-to-noise ratio. This is the particular case of C L-edge and even N K-edge. However, for O K-edge and F K-edge with energy edge around 500 and 600 eV, respectively, the more effective method to record NEXAFS spectra is using the fluorescence detector, because the X-ray emission is more probable than the electron emission. The typical NEXAFS spectra are characterized by separate peaks, i.e., the bonding-to-antibonding transitions of σ→π* or σ→σ* character. The area of each peak is proportional to the concentration of bonds, and the edge jump for any particular elemental K-edge is proportional to the total concentration of that element in the sample investigated. Notice, however, that both of these quantities, the near-edge and the edge jump, are dependent on the incident and the emission angles. For the oxygen case, the typical energy of the π* and σ*, peaks are around (530.0 ± 0.05) eV and (540.0 ± 0.05) eV, respectively, following previous well-established studies [44,45,46,47]. The presence in Figure 8 of both peaks with π* and σ* characteristics, suggests that a significant concentration of C=O and C–O bonds are present on the PS surfaces after the O_2_ plasma treatments. More interesting, due to the high-energy resolution O K-edge spectra, a close oxygen functional assignment can be accomplished.

Comparing the NEXAFS spectra of Figure 8 with model compounds O K-edge NEXAFS spectra already published for p-benzoquinone, hydroquinone, and phenols together with those of ketones [48], we can define more precisely the surface functionalization of PS surface due to the O_2_ plasma treatment. There are two main peaks within the π* region of the O K-edge spectra, at (530.0 ± 0.05) eV and (532.0 ± 0.05) eV, which have been previously reported as O 1s→π* transition in benzoquinones and cyclohexane ketones, respectively. Both transitions involve C=O bonds; however, the first one is related to aromatic carbon cycles whereas the second is related to a carbonyl bond inserted in non-aromatic carbon cycles. On the other side, the broad peak around 540.0 eV ± 0.05 can be assigned to O 1s→σ* transitions. Notice that this peak is composed of several components that are impossible to separate because their energies are very close. However, a distinctive contribution can be clearly identified centered at (535.5 ± 0.05) eV, which can be mainly assigned to the presence of C–OH bonds at the PS surfaces [48].

**Figure 9:**
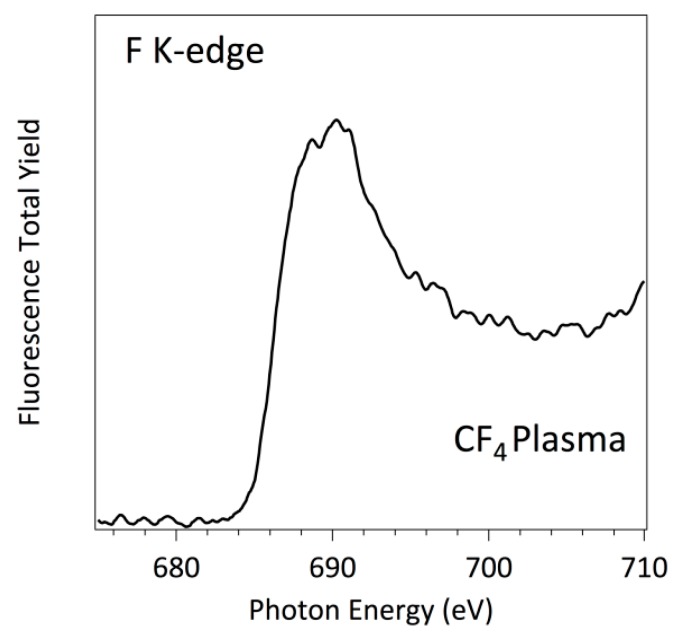
F K-edge NEXAFS spectrum of PS sample treated with CF_4_ plasma.

Secondly, using NEXAFS, we have studied the chemistry and bond structure of polystyrene surface modified by CF_4_ plasma. The F K-edge NEXAFS spectrum in Figure 9 further confirms the evidence of C–F_2_ bond induced by the CF_4_ cold gaseous plasma immersion of the PS substrate before the incubation of the albumin. The NEXAFS F K-edge spectrum in Figure 9 is dominated by a broad peak around 689 eV, with a full width at half maximum (FWHM) of ≈6 eV. This spectrum can be compared to graphite fluoride (CF*_x_*, *x* ≈ 1.1) [49], which shows two clear peaks at (689.6 ± 0.05) eV and (692.8 ± 0.05) eV. These peaks have been identified as originating from the F 1s electron transitions π*(C–F) and σ*(C–F) [50,51]. Actually, the existence of a broad peak convoluting the F 1s→π*and F 1s→σ* transitions confirms that the CF_4_ plasma treatment of the PS surface retains almost unaltered the aromatic character of the polystyrene after the plasma treatment. Damage of the aromatic bonding of the PS would be evidenced by the decreased of the F 1s→π* transition characteristic of the (C–F) bonding in aromatic carbon rings and the increase of the F 1s→σ* transition, associated with (C–F) bonding in a non-aromatic carbon ring.

In summary, Figure 8 and Figure 9 represent the spectra at oxygen K-edge for polystyrene samples treated with oxygen and fluorine plasma, which are in full agreement with the XPS measurements (Figure 3 and Table 1) on the samples. In particular, fluorine is a better oxidant than oxygen, so the surface becomes saturated with stable fluorine rich functional groups upon plasma treatment, such that no formation of oxygen functional groups occurs upon exposure to air. However, both plasma treatments functionalize the polystyrenes surfaces differently [47,52]. Koprinarov et al. found large differences in the angular dependent spectra for untreated oxygen-containing polymers, but these differences vanished after prolonged treatment with oxygen plasma [47]. Although the authors did not give an exact explanation for this observation, we believe it is due to the change of surface morphology upon treatment of such polymers with oxygen plasma. Specifically, oxygen-rich polymers like PET undergo morphological changes upon treatment with oxygen plasma [9]. Certainly, no fluorine was observed in the oxygen plasma treated sample, but a distinctive peak at F K-edge was observed for the sample treated by CF_4_ plasma (Figure 9). Seki et al. performed systematic research on F K-edge NEXAFS of various C–F systems and found rich structures [53]. In our case, the structure is pronounced but broad including both transitions with F 1s→π* and F 1s→σ* characteristics. This could be explained by the variety of functional groups obtained on the surface of PS polymer upon treatment with CF_4_ plasma, in agreement with the XPS results presented in Figure 4c, which shows various surface functionalities. The spectra presented in Figure 8 and Figure 9 indicate the presence of the functional groups on plasma-treated polystyrene determined by the O_2_ and CF_4_ plasma treatments. As the typical depth analysis of the NEXAFS detected using fluorescence is of the order of 100 nm, we only could conclude that the PS surface is functionalized throughout, within at least a surface layer region of this depth.

**Figure 10:**
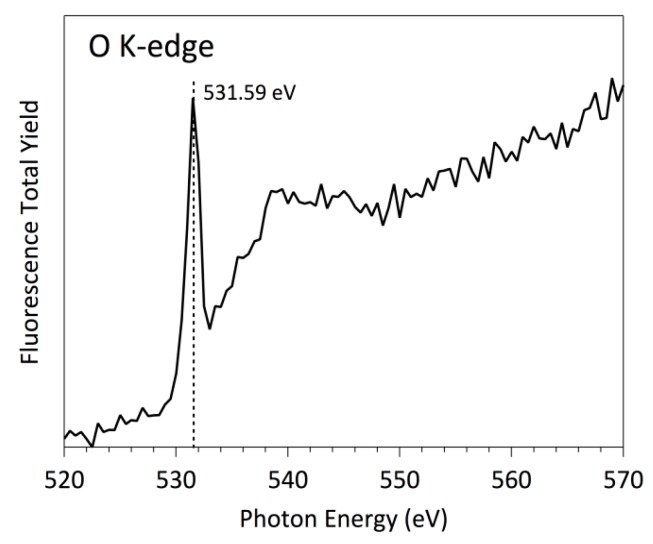
O K-edge NEXAFS spectrum of albumin incubated sample treated with oxygen plasma.

**Figure 11:**
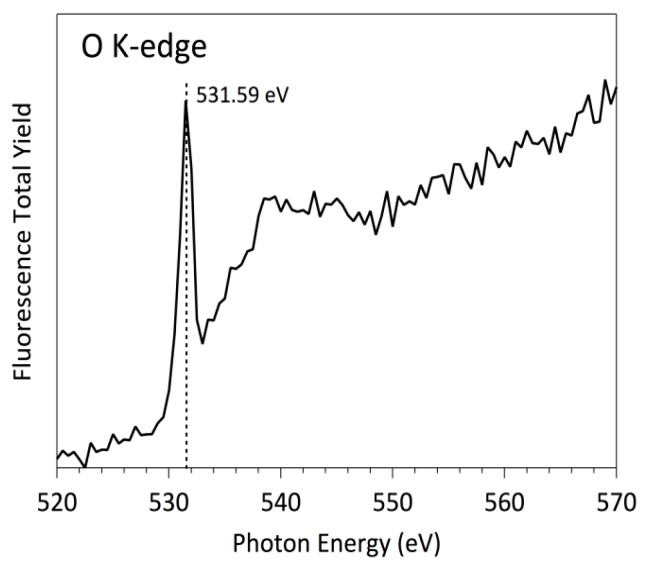
O K-edge NEXAFS spectrum of albumin incubated sample treated with CF_4_ plasma.

More pronounced variations were found when the substrates were incubated with albumin. Figure 10 and Figure 11 represent the O K-edge NEXAFS spectra of the albumin incubated sample treated with oxygen and CF_4_ plasma, respectively. The spectra were captured with short period of illumination, they show a well-defined peak at (531.6 ± 0.05) eV, which is not related to any plasma-derived functions described previously. In fact, this peak is a well-known albumin-related feature in both differently pre-treated surfaces, which can be unambiguously assigned to a carboxylic and/or amidic peak. The transition corresponds to a selective excitation of the O 1s→π* of the C=O bond in a –C(=O)–OH and/or –C(C=O)–NH_2_ chemical functions [44,45,46,54]. As expected, these spectra are similar to previously measured spectra of pristine amino acids and peptides. Further, the spectra of the individual proteins are reasonably similar to each other, which is understandable, taking into account a large amount of the individual amino acid residues constituting these proteins. Within the simplest building block approach, the spectrum of a protein may be represented by a weighted sum of the spectra of its constituent amino acids. Nevertheless, the spectrum of a peptide is undoubtedly a function of the spectra of the constituent amino acids, although not exactly a simple linear combination thereof. For the particular case of the human serum albumin, a protein with relatively typical amino acid content in terms of the ratio of aromatic to non-aromatic amino acids, the main peak can be surely assigned to an amide bond, in excellent agreement with the estimated NEXAFS spectra of peptides. Generally speaking, without taking into account a more precise approach adding a correction due to the “peptides bond formation“, the peptide bond can be evidenced in NEXAFS simply by the loss of the carboxylic acid functional group and the formation of the amide group. The effect of the O 1s peptide bond in the NEXAFS spectrum is a differential shape consisting of a small shift in the location of the O 1s→π* transitions of the C=O bonds, which is typically at (532.3 ± 0.05) eV in amino acids and at (532.0 ± 0.05) eV in peptides.

After controlled X-ray doses, a huge difference is observed in the O K-edges for both surface types: the rather sharp peak at (531.5 ± 0.05) eV, is split into a small peak at (530.7 ± 0.05) eV and a broad peak around (534.0 ± 0.05) eV. This observation is manifested in both oxygen and CF_4_ plasma-treated samples and is reproducible. Thus, it was concluded that the soft X-ray irradiation causes irreversible modification of the protein.

**Figure 12:**
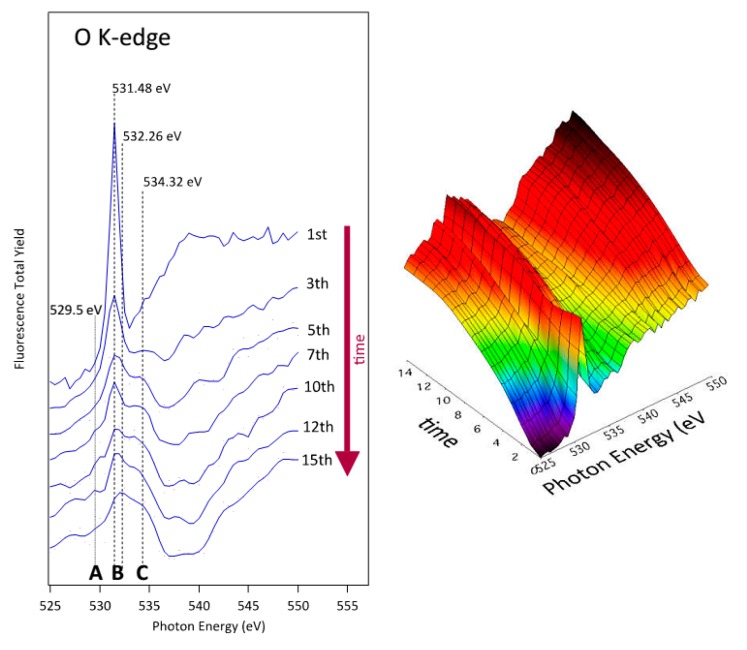
Series of O K-edge NEXAFS spectra of albumin-incubated sample treated with CF_4_ plasma and incubated with albumin (1000 s). Each spectra required irradiation for 150 s, which corresponds to one dose (see text).

**Figure 13:**
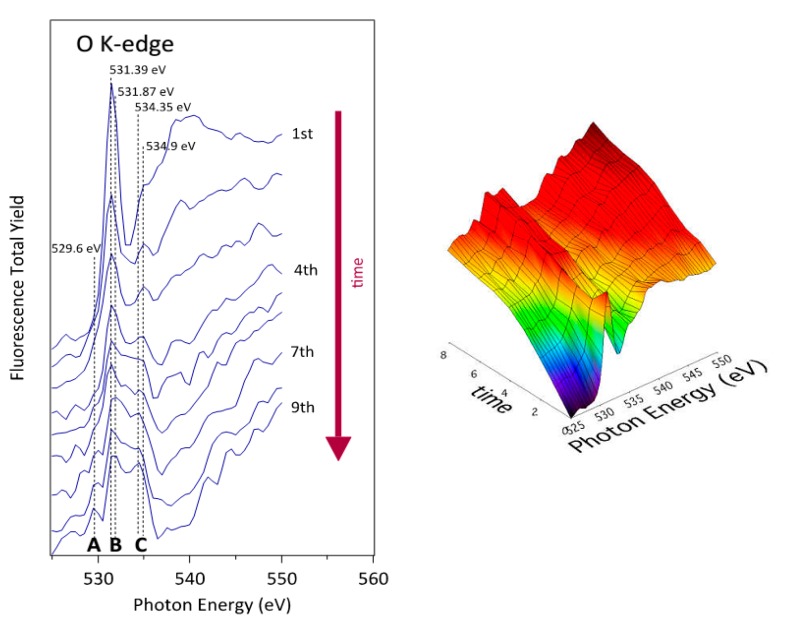
Series of O K-edge NEXAFS spectra of albumin-incubated sample treated with oxygen plasma and incubated with albumin (1000 s). Each spectra required irradiation for 150 s, which corresponds to one dose (see text).

Systematic measurements were performed in order to obtain insights into the influence of increasing irradiation doses on the albumin structure. The measurement parameters were set as follows: photon energy range of 525–550 eV, number of steps 50 and 3 s integration time. As the acquisition of each spectrum requires 150 seconds, we define this period of time as one dose of x-ray, being 1.1 × 10^14^ photons/dose, the number of photons received by the sample, during a typical NEXAFS spectrum acquisition. Using these conditions, several spectra were recorded at the same spot which are shown in Figure 12 and Figure 13 for CF_4_ and O_2_ plasma-treated substrates, respectively.

The lowest curve in Figure 12 represents the first spectrum acquired at the selected spot on the CF_4_ plasma-treated PS coated with albumin. Since no oxygen was detected on this substrate after plasma treatment (Table 1), all oxygen is bonded to albumin. The main observable effect of the increased radiation doses is that the sharp peak at (531.5 ± 0.05) eV and a broad barely distinguished peak at about (540.0 ± 0.05) eV are strongly affected. The intensity of the sharp peak is noticeably reduced and other peak starts to growth at (534.35 ± 0.05) eV. After the first radiation exposure (approximately after the fourth X-ray dose), intensity between these two peaks also starts to increase. The final NEXAFS spectra are characterized by the structures “A”, “B”, and “C” as indicated in Figure 12 and Figure 13. These fine NEXAFS structures are related to the O 1s→π* of quinone –C=O, carboxylic acid, amide and phenolic –C–OH bonds, respectively. Probably, peak “C” can also be related to carbonyl functions produced, as the carboxylic and phenolic groups, by the destruction of the amide functions characteristic of the albumin protein. The broad initial peak centered at (540.0 ± 0.05) eV is also strongly affected. Its intensity associated to the O 1s→σ* transition decreases, disappearing after the fourth radiation dose [18,55,56].

Even the second measurement performed at the same position reveals a modification of the spectrum. While the peak at (531.5 ± 0.05) eV still persists, the broad peak at (540 ± 0.05) eV is barely distinguished. The modification can be attributed directly to the destruction of the main chemical functionalities of the albumin protein mainly based on the amide group due to the illumination with X-rays or to the heat liberated by such radiation, taking into account that albumin is particularly sensitive to the denaturalization induced by heat. The modification becomes even more pronounced with further doses. Fifteen spectra were collected at the same spot to study the spectrum evolution upon irradiation. The results clearly indicate the diminishing of the broad peak at (540.0 ± 0.05) eV as well as the slow degradation of the peak at (531.5 ± 0.05) eV. These peaks are eventually replaced by a broad feature centered at about (532.0 ± 0.05) eV. Summarizing and taking into account the available peak assignments, one can conclude that both the O 1s→π* and O 1s→σ* transitions are affected by the destruction of the amide group. Albumin contains a variety of amino acids and the abundance of each amino acid varies substantially among different sources. Therefore, any discussion of selective destruction of specific amino acids upon irradiation with soft X-rays is beyond the current knowledge. However, we can indicate that the main moieties produced by the amide destruction are mostly characterized by quinone –C=O, carboxylic acid, amide and phenolic –C–OH chemical functions, and probably –C=O associated to aldehydes, with a broad peak present between peaks “B” and “C” of NEXAFS spectra shown in Figure 12 and Figure 13. Still, the most sensitive functionalities of the albumin on O_2_ and CF_4_ plasma surfaces can be compared in Figure 5 as well as in Table 1. Finally, based on analysis of the products of the protein degradation in Figure 12 and Figure 13, it can be noticed that the peak “A” at 529.5 eV distinctive of the quinone C=O functions is present only on those surfaces treated with O_2_ plasma and missing on those surfaces treated with CF_4_ plasma, which suggests that this structure probably originates from the plasma-treated polystyrene substrate rather than albumin.

The XPS peak at 291.5 eV ± 0.05 attributed to the CF_2_ functional group vanishes completely upon intensive irradiation (Figure 5b). Surprisingly, the fluorine peak in the overview spectrum persists even after irradiation (Figure 7). In fact, the concentration of fluorine in the irradiated sample is almost the same as before irradiation (Table 1). All these facts indicate reorganization of the functional groups within the thin film. It is known that X-ray radiation may cause breakage of the CF*_x_* bonds and release of fluorine [26]. The fluorine groups are buried beyond the albumin layer so it is unlikely to escape from the sample. Instead, they probably react with albumin-forming bonds like CH_2_CHF and CHF. These functionalities are already found in samples without the albumin coating and their concentration probably increases upon irradiation. They could not be revealed in Figure 5b since the corresponding peaks overlap with C–O and C–N peaks, which are dominant. Still, the broadness of the right-most peak (assigned as “C–C” in Figure 5b) increased after irradiation and this might be the consequence of albumin fluorination. The fluorination appears at the expense of the nitrogen functionalities. Although XPS overview spectra do not give precise determination of the surface film composition, the drop in the N concentration (Table 1) supports the hypothesis of partial fluorination of the albumin film upon treatment with X-rays. Namely, the difference in nitrogen concentration (7 at % versus 11 at %) is beyond the limits of the experimental error.

The decay of the peak corresponding to the π*_amide_ assignment observed in Figure 12 is consistent with the substantial decrease of the N–C=O peak in Figure 5b. Since this functional group is not present in amino acids but is formed upon joining two amino acids to form a dipeptide, one can launch a hypothesis that the destruction of peptide bonds is an important mechanism of albumin modification while irradiating with X-rays. Such destruction occurs also in the case of albumin deposited onto polystyrene samples exposed to oxygen plasma. In this case, the NEXAFS spectra in Figure 13 are similar to the NEXAFS spectra of albumin on fluorinated substrates in Figure 12. Also, the XPS results in Figure 5a show a substantial decrease of the N–C=O peak. The C–C peak, however, does not broaden after irradiation and this observation is explained by the lack of fluorine, which is, according to the above hypothesis, responsible for broadening the peak. A drop in the peak (or, rather, shoulder) assigned as “C–O, C–N” in Figure 5a should be attributed to the loss of nitrogen upon irradiation. Table 1 indicates a substantial decrease of the nitrogen concentration (from 16 to 12 at. %) upon irradiation with X-rays. The structural changes, which are revealed from NEXAFS results in Figure 13, are therefore well illustrated in the XPS results.

Finally, let us discuss the applicability of both complementary techniques (XPS and NEXAFS) for characterization of our samples. X-ray photoelectron spectroscopy is a highly surface sensitive technique since the information about the composition and structure is limited to the surface film from which photoelectrons are emitted without scattering—few nm for most organic materials. The near-edge X-ray absorption fine structure detects a fluorescent photon, which has one or two orders of magnitude larger path in solid materials than a photoelectron, so this technique gives information averaged over a thicker film. In NEXAFS, the final state of the photoelectron may be a bound state such as an exciton so the photoelectron itself need not be detected. NEXAFS therefore gives information over all possible final states of the photoelectrons. From this point of view NEXAFS has a stronger analytical power than XPS providing the X-rays do not damage the original structure of the sample. If we compare Figure 5 with Figure 12 and Figure 13, we clearly see that the modifications of the XPS signal are much weaker than the NEXAFS signal. In fact, Figure 12 and Figure 13 clearly show the destruction of the amide group upon irradiation with X-rays while the XPS results in Figure 5 only show some modifications that are difficult to explain due to overlapping of the peaks corresponding to different functional groups. On the other hand, XPS is a readily available technique since it employs a classical monochromatized source of X-rays, while NEXAFS is feasible only at synchrotrons where intensive sources of X-rays with scalable energy are available. In the case of organic materials characterization, one should, however, note the destruction of sample upon irradiation with X-rays.

## 4. Conclusions

Two complementary methods (NEXAFS and XPS) for thin film characterization were applied in order to study the destruction of albumin thin films on polymer substrates upon irradiation with soft X-rays. NEXAFS results showed a rapid drop of the O1s π*_COO_ assignment and a gradual decrease of the π*_amide_ assignment. The composition of the albumin film changed after the irradiation and the most significant effect was the depletion of nitrogen. A substantial difference between samples pre-treated with CF_4_ and O_2_ plasmas was explained by different mechanisms involved. In the case of fluorinated polystyrene, the X-rays destroy fluorine-rich functional groups and cause the formation of functionalities such as CH_2_CHF. When polystyrene substrates were functionalized with oxygen groups prior to incubation with albumin, the NEXAFS results obtained at oxygen K-edge manifested similar structural charges but without fluorination. In both cases, prolonged irradiation caused substantial destruction of the peptide bonds as illustrated by both methods.

## Figures and Tables

**Table 1:** Concentration of elements as determined by XPS (in at %).

Sample	C	O	N	F
Untreated PS	100			
Pure albumin	66	19	15	
PS + O_2_ plasma	73	27		
PS + CF_4_ plasma	43	1		56
PS + O_2_ plasma + albumin prior irradiation	66	18	16	
PS + O_2_ plasma + albumin after irradiation	68	20	12	
PS + CF_4_ plasma + albumin prior irradiation	64	15	11	10
PS + CF_4_ plasma + albumin after irradiation	72	12	7	9

## Abbreviations

The following abbreviations are used in this manuscript:

AFM	Atomic force microscopy
FWHM	Full width at half maximum
HAS	Human serum albumin
LUMO	Lowest unoccupied molecular orbital
NEXAFS	Near-edge X-ray absorption fine structure
PS	Polystyrene
RF	Radiofrequency
UPS	Ultraviolet photoelectron spectroscopy
UV	Ultraviolet
VUV	Vacuum ultraviolet radiation
XANES	X-ray absorption near-edge spectroscopy
XPS	X-ray photoelectron spectroscopy
